# The correlation between vaginal pathogens and high-risk human papilloma virus infection: a meta-analysis of case-control studies

**DOI:** 10.3389/fonc.2024.1423118

**Published:** 2024-11-21

**Authors:** Jun Yang, Xin Long, Sijing Li, Min Zhou, Li-Na Hu

**Affiliations:** ^1^ Department of Obstetrics and Gynecology, The Second Affiliated Hospital of Chongqing Medical University, Chongqing, China; ^2^ Department of Obstetrics and Gynecology, Women and Children’s Hospital of Chongqing Medical University, Chongqing, China; ^3^ Department of Obstetrics and Gynecology, Chongqing Health Center for Women and Children, Chongqing, China; ^4^ The Center for Reproductive Medicine, Obstetrics and Gynecology Department, The Second Affiliated Hospital of Chongqing Medical University, Chongqing, China; ^5^ Joint International Research Lab for Reproduction and Development, Ministry of Education, Chongqing, China; ^6^ Reproduction and Stem Cell Therapy Research Center of Chongqing, Chongqing, China

**Keywords:** HR-HPV, vaginal microbiota, vaginal pathogens, bacterial vaginosis, vaginal infection, meta-analysis

## Abstract

**Background:**

Systematic study on the relationship between vaginal microbiota and high-risk human papillomavirus infection (HR-HPV) is limited. Hence, the aim of this study is to investigate the correlation between vaginal microbiota and HR-HPV infection through a meta-analysis of case-control studies.

**Methods:**

Chinese Journal Full-text database, Wanfang database, PubMed database, VIP Chinese Science and Technology Journal database, Web of Science, ScienceDirect, ProQuest, JSTOR, Wiley, and IEEE Xplore were synthetically searched for studies about the correlation between vaginal microbiota and HR-HPV infection. Revman 5.3 software was used to assess the relationship between vaginal microbiota and HPV infection through meta-analysis. Finally, forest map was used to calculate the results and funnel plot was applied to test the publication bias.

**Results:**

Fourteen independent studies were admitted in this study, containing a total of 21, 446 women in gynecological outpatients. Compared with HR-HPV negative group, the prevalence of bacterial vaginosis (BV) [odds ratio (OR)=2.45, 95% confidence intervals (CI): 1.83-3.27, *P*<0.00001], *Ureaplasma urealyticum* (UU) (OR=1.38, 95% CI: 1.23-1.54, *P*<0.00001), and *Chlamydia trachomatis* (CT) (OR=3.53, 95% CI: 2.82-4.41, *P*<0.00001) increased in HR-HPV positive group through meta-analysis, while, there was no significant difference in the prevalence of *trichomonal vaginitis* (TV) (OR=1.69, 95% CI: 0.97-2.96, P=0.06) and *vulvovaginal candidiasis* (VVC) (OR=0.91, 95% CI: 0.54-1.51, P=0.71.

**Conclusions:**

Vaginal pathogens are closely related to HR-HPV infection. When BV, UU, and CT are abnormal, the risk of HR-HPV infection is increased.

## Introduction

Human papillomavirus (HPV) are a large class of double-stranded DNA viruses that can infect squamous epithelial cells of the human skin layer and mucous membrane (1, 2). On the basis of different carcinogenic risks, HPV types are classified as low-risk (LR-HPV) and high-risk (HR-HPV) (3–5). Persistent infection with HR-HPV has been strongly associated with the development of cervical cancer (6–8). Cervical cancer is the fourth most common cancer among women worldwide, imposing a persistent burden on women’s health (9–11). The number of deaths due to cervical cancer is up to 266,000 globally every year, 85% of which are citizens of developing countries (12). It has been reported that continuous infection with HR-HPV for 8 to 24 months can lead to cervical intraepithelial neoplasia, which may progress to cervical cancer after 8 to 12 years (13). In line with the study, most cervical cancers are related to persistent infection with HR-HPV in the cervical mucosa (14–16). Despite the advances in the treatment of cervical cancer, the long-term prognosis of patients with metastatic, persistent, and recurrent cervical cancer still remains unsatisfactory (17, 18). Moreover, the exact risk factors and mechanisms of HPV infection and persistent infection remain unclear. In addition, it is a long process from HR-HPV infection to cervical intraepithelial neoplasia with vaginal microbiota changes (19).

The past decade has revealed a rapid advancement in our understanding of the interplay between vaginal microbiota and HPV infection. It has been reported that vaginal microbiota dysbiosis is associated with HPV infection and that vaginal microbiota dysbiosis contributes to HR-HPV persistent infection and cervical lesions (10, 19, 20). It has been reported that an imbalance of the vaginal microbiota may increase the risk of HPV infection. For example, enhanced diversity of vaginal microbiota combined with decreased relative abundance of *Lactobacillus* spp. is participated with HPV acquisition and persistence (21). At the same time, HPV infection may in turn further disrupt the balance of vaginal microbiota (22). Maintaining a suitable vaginal microbiota environment is crucial for preventing HPV infection and reducing the risk of persistent infection. However, the correlation between HPV infection and vaginal microbiota is still controversial, and there is a lack of sufficient evidence-based medical evidence and support from large-sample clinical data. Meta-analysis is a statistical method for systematic and comprehensive quantitative analysis of multiple existing and independent research results with the same research purpose (23).

Therefore, this study applied the meta-analysis to perform a statistical analysis of the published literature on the correlation between vaginal microbiota and HR-HPV infection, so as to provide theoretical guidance for the prevention and treatment of clinical HPV infection and cervical cancer.

## Methods

### Literature screening

We systematically searched for vaginal microbiota and HR-HPV infection in the Chinese Journal Full-text database, Wanfang database, PubMed database, VIP Chinese Science and Technology Journal database, Web Of Science, ScienceDirect, ProQuest, JSTOR, Wiley, and IEEE Xplore, without any language limitation. We also searched the reference in the original literature, combining subject words and free words.

The search terms were composed of the following themes: “HR-HPV,” “HPV,” “TAVR,” “high-risk human papilloma virus,” “human papilloma virus,” and so forth. We also searched the following themes: “vaginal microbiota,” “kysthitis,” “vaginal cleanliness,” “bacterial vaginosis,” “trichomonal vaginitis,” “vulvovaginal candidiasis,” “Pondus Hydrogenii,” and so forth. We adopted the search strategy of subject term + free term and adjusted the search strategy according to the database. The specific search strategy is [HR-HPV (Title/Abstract) OR HPV (Title/Abstract)] OR [high-risk human papilloma virus (Title/Abstract)] OR [human papilloma virus (Title/Abstract)] AND [vaginal microbiota (Title/Abstract)] OR [kysthitis (Title/Abstract)] OR [vaginal cleanliness (Title/Abstract)] OR [bacterial vaginosis (Title/Abstract)] OR [BV (Title/Abstract)] OR [trichomonal vaginitis (Title/Abstract)] OR [TV(Title/Abstract)] OR [vulvovaginal candidiasis (Title/Abstract)] OR [VVC(Title/Abstract)] OR [ureaplasma urealyticum (Title/Abstract)] OR [chlamydia trachomatis (Title/Abstract)].

### Inclusion and exclusion criteria

The following criteria were performed for the inclusion of eligible literature: (1) Published studies on the association between vaginal microbiota and HPV infection where Chinese literature was published in core journals, (2) the study sample size was in accordance with the research standards and the statistical methods were reasonable, and (3) the research methods of all literatures were similar. (4) For replicated studies, one of them was used; (5) there was no languages variety. The following criteria were utilized for the exclusion of improper literature: (1) review articles, duplicate literature, and unpublished literature; (2) relevant specific data could not be obtained; and (3) the group was inconsistent with the inclusion criteria of this study design.

The design of the included study also should combine the following criteria: (1) the type of study was a case-control study. Patients in the case group had a definite diagnosis of HR-HPV positive, and patients in the control group had a definite diagnosis of HR-HPV negative. (2) HR-HPV included HPV-16, 18, 31, 33, 35, 39, 45, 51, 52, 56, 58, 59, 66, and 68 (24). (3) There was no restriction on the testing of HR-HPV, and it could include single-base extension, polymerase chain reaction (PCR)—reverse dot hybridization, hybrid capture-chemiluminescence method, enzyme-linked immunosorbent assay (ELISA), *in-situ* hybridization, matrix Assisted Laser Desorption Ionization Time-of-flight mass spectrometry (MALDI-TOFMS), and so on. (4) The research data encompassed the HPV-positive group, the HPV-negative group, bacterial vaginosis (BV), *trichomonal vaginitis* (TV), *vulvovaginal candidiasis* (VVC), *Ureaplasma urealyticum* (UU), and *Chlamydia trachomatis* (CT). (5) Diagnostic criteria for vaginal microbiota dysbiosis (25): the diagnostic methods for BV were rapid BV kit, gram staining smear, or Nugent (score ≥7). The diagnostic methods of VVC were fungal culture, gram staining smear showing false mycelia and spores, or smear microscopy. The TV diagnosis method was that trichomonas vaginalis was microscopically observed in 0.9% sodium chloride injection. For the UU susceptibility test, cervical secretions were cultured in the microbial chamber, and UU routine detection and UU susceptibility identification were carried out in strict accordance with the operating instructions of the kit. CT was detected by real-time fluorescent polymerase chain reaction (PCR), RNA real-time fluorescent nucleic acid isothermal amplification, transcription-mediated nucleic acid isothermal amplification enzyme-linked immunosorbent assay, direct immunofluorescence assay or rapid immunochromatographic assay.

### Screening method and quality evaluation of the study

Research screening steps: The literature was preliminarily screened based on the title and abstract of the research. Then, the literature was ultimately obtained by reading the full text and conducting further screening in strict accordance with the inclusion design. Quality evaluation methods: The Newcastle-Ottawa-Scale (NOS) scoring standard (including selection, comparability, and exposure) was utilized to independently assess the quality of the selected literature. The total score of NOS was 9 points, and the literature quality was regarded as high when the score was ≥7. Quality control of literature screening: two researchers were responsible for formulating the search strategy, conducting literature retrieval, screening the literature, and evaluating the literature quality. In case of disagreement between the two, a third party would make the decision. Finally, the data included in the study would be collated and extracted. Data entry: The data of the included study were extracted by two researchers and collected into a pre-standardized data table. All data were revised by a third researcher to ensure data accuracy.

### Statistical analysis

The meta-analysis was performed by RevMan 5.3. The Z-test was applied to analyze the heterogeneity among the studies. When there was no heterogeneity among the studies (*P* > 0.1 and *I* ^2^ < 50%), the fixed effects model (FEM) was utilized. When statistical heterogeneity existed between studies and the source of heterogeneity could not be eliminated, the random effects model (REM) was adopted. Sensitivity was analyzed by successive exclusion of individual studies and recalculation of their combined effect sizes.

## Results

### Literature screening situation

**Figure 1:**
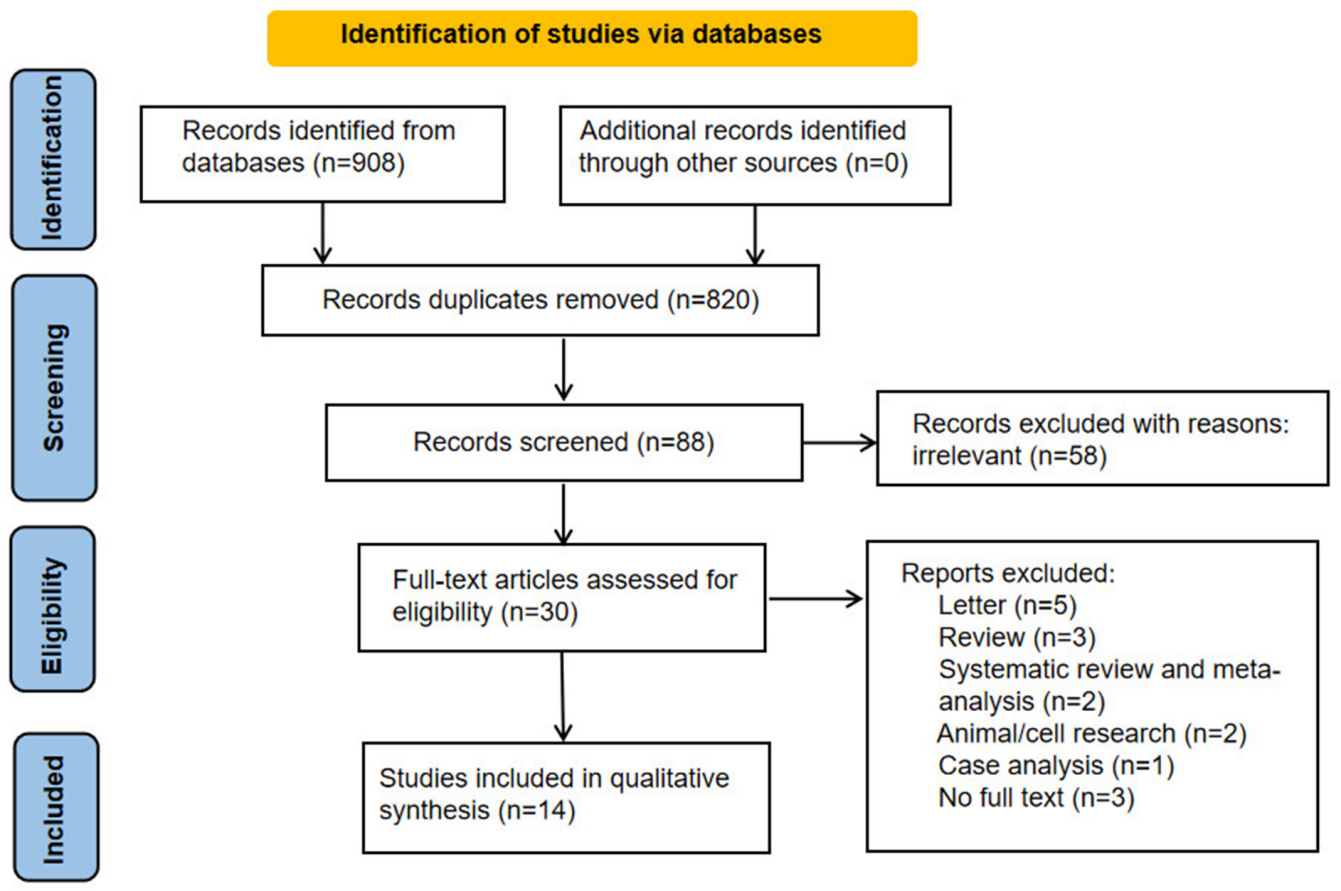
The steps of literature screening.

Ultimately, 88 works of English literatures were obtained. On the basis of the titles and abstracts of the literatures, duplicate literatures were further screened and eliminated, and a total of 30 literatures were selected. After reading the full text of the 30 literatures, 14 English literatures were finally included. A total of 21,446 women in gynecological outpatients underwent HPV and vaginal microbiota testing. The steps of the literature search are shown in **Figure 1**.

### Basic features of the 14 studies

The quality evaluation of the 14 included literatures (26–39) was all ≥7 points. The basic characteristics of the fourteen studies were shown in **Table 1**.

**Table 1:** Basic characteristics of 14 included studies.

The first author	Year	Object of study	Number of cases	BV	TV	VVC	UU	CT	Quality evaluation (point)
Lv PP (34)	2019	HR-HPV^+^	254	52	8	NR	39	40	8
		HR-HPV^−^	572	40	1	NR	49	23	
Zhang D (38)	2017	HR-HPV^+^	76	18	3	2	29	4	8
		HR-HPV^−^	877	82	13	33	309	17	
Liu JH (31)	2016	HR-HPV^+^	1452	94	55	NR	845	78	7
		HR-HPV^−^	2838	66	93	NR	1460	48	
Xu CY (37)	2012	HR-HPV^+^	622	107	9	7	NR	NR	8
		HR-HPV^−^	5590	446	53	98	NR	NR	
Verteramo R (36)	2009	HR-HPV^+^	266	23	3	32	96	37	8
		HR-HPV^−^	591	31	7	80	147	32	
da Silva CS (27)	2004	HR-HPV^+^	26	14	NR	5	NR	9	8
		HR-HPV^−^	26	4	NR	6	NR	2	
Murta EF (35)	2000	HR-HPV^+^	390	NR	7	47	NR	NR	8
		HR-HPV^−^	396	NR	9	88	NR	NR	
Lu H (33)	2015	HR-HPV^+^	1738	270	NR	NR	NR	43	7
		HR-HPV^−^	1764	54	NR	NR	NR	5	
Huang J (29)	2023	HR-HPV^+^	416	147	44	34	NR	NR	7
		HR-HPV^−^	911	190	31	32	NR	NR	
Lin WY (30)	2022	HR-HPV^+^	138	68	0	18	NR	NR	8
		HR-HPV^−^	310	120	4	36	NR	NR	
Bommana S (26)	2024	HR-HPV^+^	41	NR	NR	NR	NR	21	7
		HR-HPV^−^	217	NR	NR	NR	NR	78	
Dong BH (28)	2022	HR-HPV^+^	270	106	NR	NR	NR	NR	7
		HR-HPV^−^	550	200	NR	NR	NR	NR	
Liu YJ (32)	2023	HR-HPV^+^	271	163	NR	NR	NR	NR	7
		HR-HPV^−^	744	330	NR	NR	NR	NR	
Zhang Z (39)	2021	HR-HPV^+^	80	23	NR	NR	NR	NR	8
		HR-HPV^−^	20	2	NR	NR	NR	NR	

The symbol "+" means positive and the symbol "^−^" means negative. BV, bacterial vaginosis; TV, *trichomonal vaginitis*; VVC, *vulvovaginal candidiasis*; UU, *Ureaplasma urealyticum*; CT, *Chlamydia trachomatis*; NR, none reported.

### The combined results of the study

The 14 studies included in this study were combined. The prevalence of BV, TV, UU, and CT in the HR-HPV–positive group was 19.34%, 3.57%, 7.5%, 49.3%, and 6.02%, respectively. The prevalence of BV, TV, VVC, UU, and CT in the HR-HPV–negative group was 10.58%, 1.75%, 4.29%, 40.3%, and 2.98%, respectively.

### Correlation between vaginal microecosystem and HR-HPV infection

#### BV and HR-HPV

**Figure 2:**
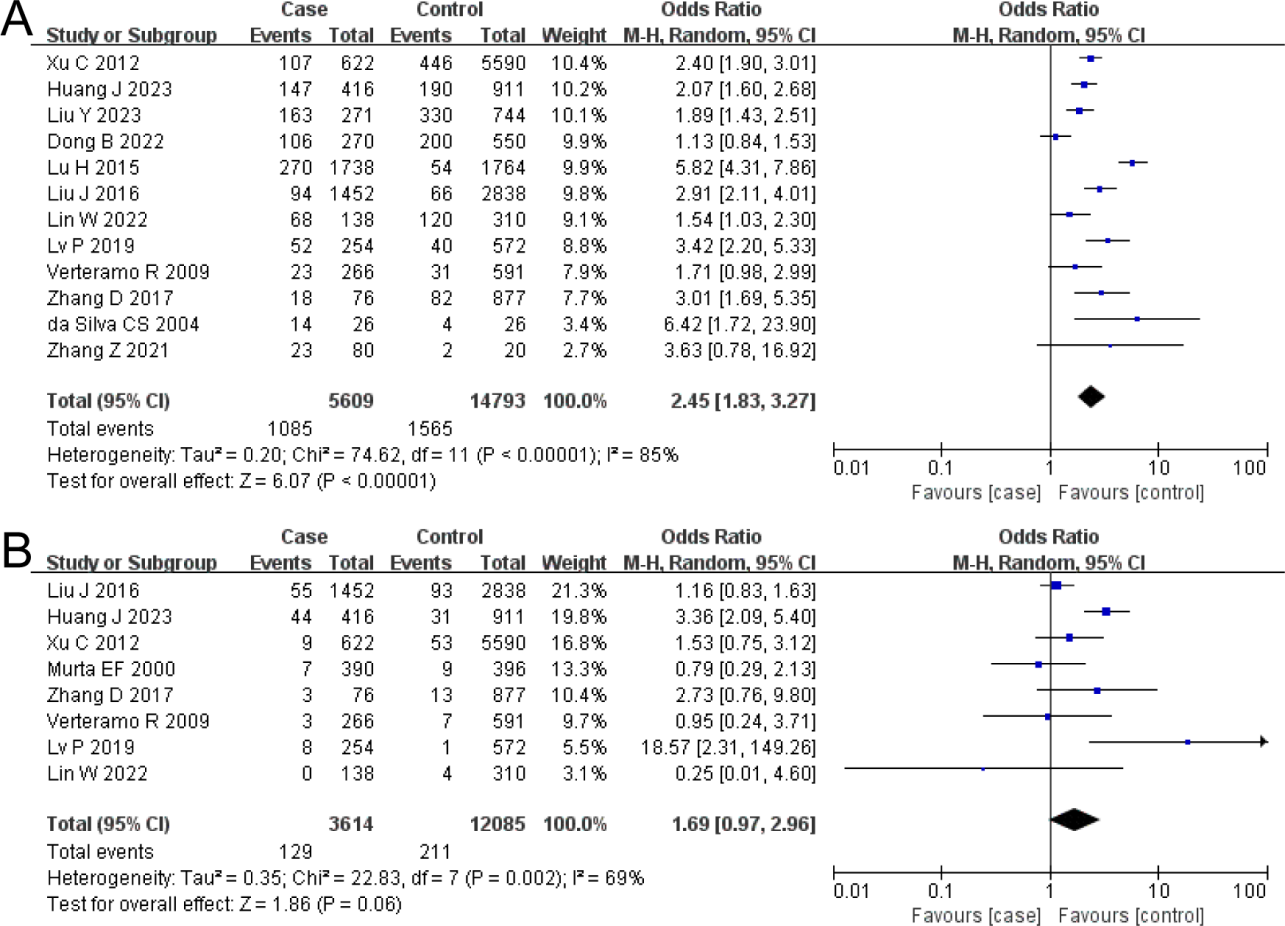
The risk association between bacterial vaginosis (BV) or *trichomonal vaginitis* (TV) and high-risk human papillomavirus infection (HR-HPV) infection. **(A)** BV; **(B)** TV.

The combined OR of BV and HR-HPV infection was 2.45 (95% CI: 1.83–3.27). The HR-HPV–positive group had a higher prevalence of BV, the difference being statistically significant (*Z* = 6.07, *P* < 0.00001), as shown in **Figure 2A**.

#### TV and HR-HPV

The combined OR of TV and HR-HPV infection was 1.69 (95% CI: 0.97–2.96). There was no significant difference in TV prevalence between the HR-HPV positive and the negative (*Z* = 1.86, *P* = 0.06), as shown in **Figure 2B**.

#### VVC and HR-HPV

**Figure 3:**
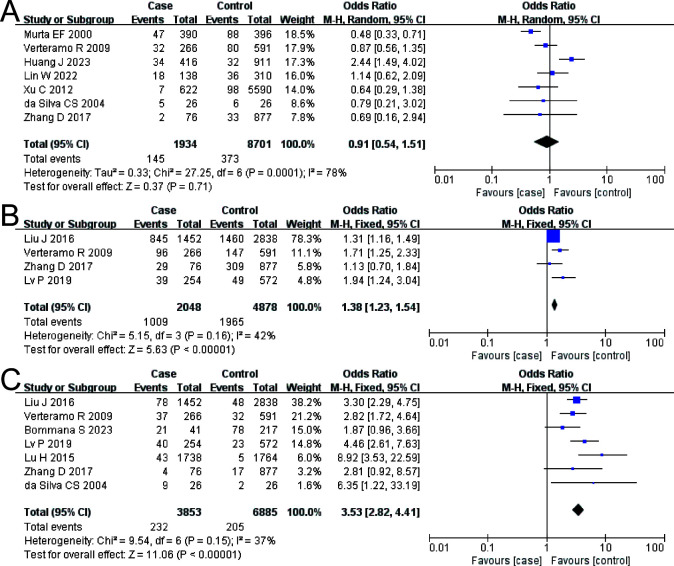
The risk association between *vulvovaginal candidiasis* (VVC), *Ureaplasma urealyticum* (UU), *Chlamydia trachomatis* (CT), and high-risk human papillomavirus infection (HR-HPV) infection. **(A)** VVC; **(B)** UU; **(C)** CT.

The combined OR of VVC and HR-HPV infection was 0.91 (95% CI: 0.54–1.51). There was no significant difference in VVC prevalence between the HR-HPV positive and the negative (Z = 0.37, *P* = 0.71), as shown in **Figure 3A**.

#### UU and HR-HPV

The combined OR of UU and HR-HPV infection was 1.38 (95% CI: 1.23–1.54). The HR-HPV–positive group had a higher prevalence of UU, the difference being statistically significant (Z = 5.63, *P* < 0.00001), as shown in **Figure 3B**.

#### CT and HR-HPV

The combined OR of CT and HR-HPV infection was 3.53 (95% CI: 2.82–4.41). The HR-HPV–positive group had a higher prevalence of CT (*Z* = 11.06, *P* < 0.00001), as shown in **Figure 3C**.

### Bias risk assessment

**Figure 4:**
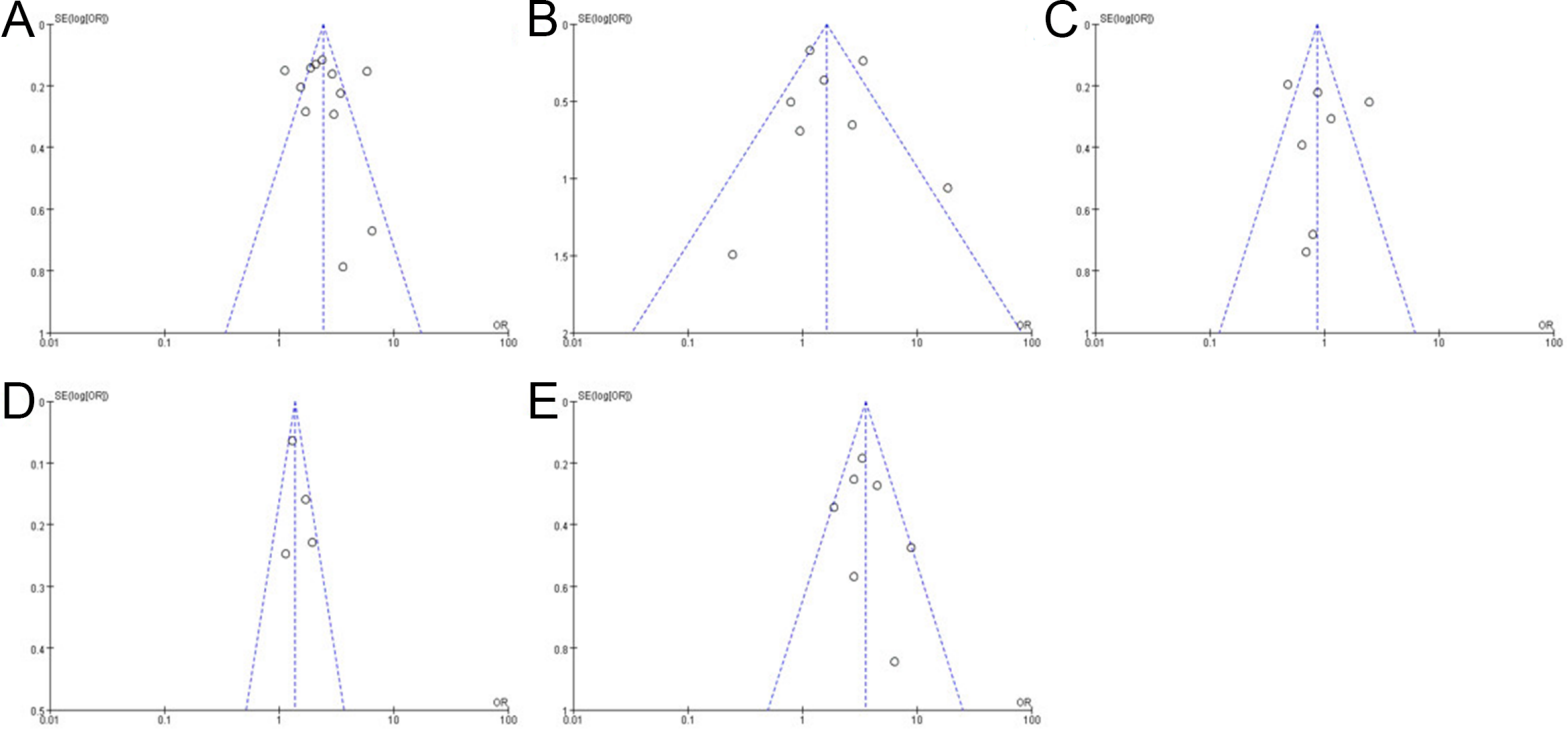
Funnel plot shows bacterial vaginosis (BV) risk **(A)**, *trichomonal vaginitis* (TV) risk **(B)**, *vulvovaginal candidiasis* (VVC) risk **(C)**, *Ureaplasma urealyticum* (UU) risk **(D)**, and *Chlamydia trachomatis* (CT) risk **(E)**.

The funnel plot was shown in **Figure 4**, which reveals that some studies intersect or are outside the funnel slope, indicating that these studies may have the risk of bias, which might be related to the small number of studies included in this study.

## Discussion

Once HPV infection, it escapes or avoids host immune response (40, 41). During epithelial cell differentiation, HPV infection results in cell growth and increases virus-mediated immune escape ability (42, 43). On the other hand, it can cause specific interference with innate and adaptive immunity through its own concealment, thereby causing persistent infection in the host (7). Previous studies have confirmed that persistent HR-HPV infection can lead to the development of cervical cancer (7, 44). Compared to normal vaginal flora, Bacteroidetes and Fusobacteria increased in HR-HPV–infected group (45). Moreover, it has been reported that BV, TV, and VVC were significantly correlated with HR-HPV infection (6). For instance, women with persistent HR-HPV had a prevalence of BV of 11%, while women who cleared their HR-HPV only had 5% (46). However, not all findings support this correlation. Therefore, it is necessary to systematically evaluate the correlation between vaginal microbiota and HR-HPV infection using evidence-based medicine methods.

Our meta-analysis results indicated that the prevalence of BV, UU, and CT was significantly higher in HR-HPV–positive patients than that in HR-HPV–negative patients, while the prevalence of VVC and TV had no significant difference. Our results suggested that BV, UU, and CT are risk factors for HR-HPV infection, while VVC and TV have no significant correlation with HR-HPV infection. The results of the bias risk assessment showed that each funnel plot had good symmetry and the risk of bias was considered to be small, indicating that the conclusions of this study had certain stability and reliability.

There are various kinds of female vaginal microflora, which are symbiotic and antagonistic with each other and in a state of dynamic equilibrium under normal conditions. Abnormalities in the reproductive tract system’s anatomical structure, local immunity, microbial flora, endocrine, and other factors can cause microbial flora imbalance (47). The normal vaginal microecological state (48) is as follows: the dominant bacteria of the vagina is *Lactobacillus*, the concentration of bacteria is grades II to III, the diversity is grades II to III, the pH value is 3.8 to 4.5, the leucocyte esterase is negative, and the *Lactobacillus* function is normal (normal secretion of H_2_O_2_). Once the vaginal microbiota is unbalanced, various types of vaginitis, such as BV and TV, will occur (49). BV is a mixed endogenous infection caused by a significant reduction in the vaginal production of hydrogen peroxide-producing *Lactobacillus* and the proliferation of *Gardnerella* and anaerobic bacteria. It has been reported that there is a significant correlation between BV and HR-HPV infection (50), and our study has also confirmed this result. *Lactobacillus* spp. are thought to confer protection against HPV infection (51, 52). BV is a proinflammatory genital condition characterized by high vaginal bacterial diversity and a paucity of *Lactobacillus* spp. (53). Moreover, the increase of mucin-degrading enzyme in vaginal fluid may destroy the glial layer on the surface of cervical epithelial cells, thus making the body more vulnerable to virus invasion (54). BV may also lead to a decrease in the secretion of secreted leukocyte protease inhibitor in the vagina, thereby weakening the body’s ability to inhibit the virus (30); simultaneously, it may reduce the secretion of IL-1β and other cytokines in the cervical epithelium, thus disrupting the immune balance of the body and making the body easily infected with HR-HPV (55, 56). TV is a type of lower genital tract inflammation caused by *Trichomonas vaginalis* infection. *Trichomonas* consumes glycogen in vaginal epithelial cells and competitively inhibits the growth of *Lactobacillus*, resulting in an increased vaginal pH and frequent co-infection with BV (57). This inflammation was once regarded as the cause of cervical cancer, which may cause degeneration and necrosis of the cervical epithelium, causing damage to the barrier function of the cervical epithelium, thereby triggering an immune response and releasing inflammatory factors, creating favorable conditions for the reproduction of other pathogenic microorganisms, thereby promoting the persistent infection of HPV, and ultimately promoting the occurrence of cervical lesions (58). Some scholars believe that TV has a certain correlation with HPV infection. They found that the HPV-16 infection rate of TV patients was 6.5 times that of TV-negative patients (59). The reason maybe that the patient was infected with TV after the vaginal mucosa congestion and edema, the vaginal and cervical mucosa were damaged. The conclusions of the meta-analysis of BV and TV in this study were consistent with the majority of published studies (60). However, there was no significant correlation between VVC and HR-HPV infection in our study, which reveals different results compared with previous studies. This needs to be further studied in future research. Vaccination against HPV can prevent HPV infection, thereby preventing diseases associated with HPV infection, such as cervical cancer, anal cancer, vulvar cancer, and vaginal cancer. Nevertheless, it cannot prevent all types of vaginitis. HPV is a virus that can cause a variety of diseases, the most common of which is cervical cancer (61). HPV vaccines can assist people in preventing HPV infection, thereby reducing the risk of developing diseases related to HPV infection. However, vaginitis is caused by microorganisms such as bacteria and fungi, and the HPV vaccine is unable to prevent infection by these microorganisms. However, considering the findings of this study that BV and abnormal vaginal pH are associated with HR-HPV infection and that HR-HPV infection may promote the occurrence of BV and abnormal vaginal pH. Therefore, we believe that vaccination against HPV may reduce the occurrence of BV and abnormal vaginal pH. The HPV vaccine has a significant potential impact on vaginal microbiota and the prevention of HPV infection. In terms of preventing HPV infection, the HPV vaccine establishes a defense line against HPV virus invasion for the human body by inducing the body to produce specific antibodies (62). This greatly reduces the risk of HPV infection. The effect is more obvious, especially for those who have not had sexual intercourse or have infrequent sexual intercourse. In the long term, it can significantly reduce the incidence of malignant diseases such as cervical cancer and anal cancer caused by persistent HPV infection (63). For vaginal microbiota, HPV infection may lead to vaginal flora imbalance and destroy the microecological balance (64). After vaccination with HPV vaccine, the possibility of HPV infection is reduced, which helps maintain the normal microbial community in the vagina (63). A healthy vaginal microbiota can enhance the self-cleaning ability of the vagina and resist the invasion of pathogens. In addition, a stable microecological environment is also conducive to maintaining the normal physiological functions of the female reproductive system and reducing the occurrence of diseases such as vaginitis and cervicitis.

## Conclusion

Vaginal pathogens are closely related to HR-HPV infection. BV, UU, and CT are high-risk factors for HR-HPV infection. Patients with high-risk factors should be vigilant, and timely preventive interventions should be taken to reduce the risk of HR-HPV infection. This study is an update meta-analysis of the research on the correlation between vaginal microbiota and HR-HPV infection. The risk of research bias was low, and the conclusions were stable and reliable to a certain extent, which could guide the clinical prevention, control, and treatment of HR-HPV. However, due to the limited number of studies included in this study, the risk of research bias could not be disregarded. In addition, incomplete data reporting of some vaginal pathogens may affect the comprehensiveness and accuracy of the detection results. Therefore, in subsequent studies, we need to further increase the inclusion of studies and improve data collection and analysis to enhance the reliability of the results of this study.

## Data Availability

Publicly available datasets were analyzed in this study. Manuscript contains all data.

## References

[1] Baumgarth N Szubin R Dolganov GM Watnik MR Greenspan D Da Costa M . Highly tissue substructure-specific effects of human papilloma virus in mucosa of HIV-infected patients revealed by laser-dissection microscopy-assisted gene expression profiling. Am J Pathol. (2004) 165:707–18. doi: 10.1016/S0002-9440(10)63334-2 PMC1618607 15331396

[2] Colpani V Soares Falcetta F Bacelo Bidinotto A Kops NL Falavigna M Serpa Hammes L . Prevalence of human papillomavirus (HPV) in Brazil: A systematic review and meta-analysis. PloS One. (2020) 15:e0229154. doi: 10.1371/journal.pone.0229154 32084177 PMC7034815

[3] Alotaibi M Valova V T HA Stromberger C Kofla G Olze H . Impact of smoking on the survival of patients with high-risk HPV-positive HNSCC: a meta-analysis. In Vivo. (2021) 35:1017–26. doi: 10.21873/invivo.12345 PMC8045080 33622897

[4] Aziz H Iqbal H Mahmood H Fatima S Faheem M Sattar AA . Human papillomavirus infection in females with normal cervical cytology: Genotyping and phylogenetic analysis among women in Punjab, Pakistan. Int J Infect Dis. (2018) 66:83–9. doi: 10.1016/j.ijid.2017.11.009 29138009

[5] Kudela E Liskova A Samec M Koklesova L Holubekova V Rokos T . The interplay between the vaginal microbiome and innate immunity in the focus of predictive, preventive, and personalized medical approach to combat HPV-induced cervical cancer. EPMA J. (2021) 12:199–220. doi: 10.1007/s13167-021-00244-3 34194585 PMC8192654

[6] Kyrgiou M Mitra A Moscicki AB . Does the vaginal microbiota play a role in the development of cervical cancer? Transl Res. (2017) 179:168–82. doi: 10.1016/j.trsl.2016.07.004 PMC5164950 27477083

[7] Paehler Vor der Holte A Fangk I Glombitza S Glombitza S Wilkens L Welkoborsky HJ . Impact of human papillomaviruses (HPV) on recurrence rate and Malignant progression of sinonasal papillomas. Cancer Med. (2021) 10:634–41. doi: 10.1002/cam4.3642 PMC7877357 33350606

[8] Peng Q Wang L Zuo L Gao S Jiang X Han Y . HPV E6/E7: insights into their regulatory role and mechanism in signaling pathways in HPV-associated tumor. Cancer Gene Ther. (2024) 31:9–17. doi: 10.1038/s41417-023-00682-3 38102462

[9] Begliarzade S Sufianov A Ilyasova T Shumadalova A Sufianov R Beylerli O . Circular RNA in cervical cancer: Fundamental mechanism and clinical potential. Noncoding RNA Res. (2024) 9:116–24. doi: 10.1016/j.ncrna.2023.11.009 PMC10686810 38035041

[10] Castanheira CP Sallas ML Nunes RAL Lorenzi NPC Termini L . Microbiome and cervical cancer. Pathobiology. (2021) 88:187–97. doi: 10.1159/000511477 33227782

[11] Zhang C Yuan L Zou Q Shao C Jia Y Li J . CircMAST1 inhibits cervical cancer progression by hindering the N4-acetylcytidine modification of YAP mRNA. Cell Mol Biol Lett. (2024) 29:25. doi: 10.1186/s11658-024-00540-6 38331765 PMC10854152

[12] Huang H Feng YL Wan T Zhang YN Cao XP Huang YW . Effectiveness of sequential chemoradiation vs concurrent chemoradiation or radiation alone in adjuvant treatment after hysterectomy for cervical cancer: the STARS phase 3 randomized clinical trial. JAMA Oncol. (2021) 7:361–9. doi: 10.1001/jamaoncol.2020.7168 PMC7809615 33443541

[13] Arbyn M Simon M Peeters E Xu L Meijer C Berkhof J . 2020 list of human papillomavirus assays suitable for primary cervical cancer screening. Clin Microbiol Infect. (2021) 27:1083–95. doi: 10.1016/j.cmi.2021.04.031 33975008

[14] Fitzpatrick M Pathipati MP McCarty K Rosenthal A Katzenstein D Chirenje ZM . Knowledge, attitudes, and practices of cervical Cancer screening among HIV-positive and HIV-negative women participating in human papillomavirus screening in rural Zimbabwe. BMC Womens Health. (2020) 20:153. doi: 10.1186/s12905-020-01017-2 32711530 PMC7382027

[15] Kori M Arga KY Mardinoglu A Turanli B . Repositioning of anti-inflammatory drugs for the treatment of cervical cancer sub-types. Front Pharmacol. (2022) 13:884548. doi: 10.3389/fphar.2022.884548 35770086 PMC9234276

[16] Randall TC Ghebre R . Challenges in prevention and care delivery for women with cervical cancer in sub-Saharan Africa. Front Oncol. (2016) 6:160. doi: 10.3389/fonc.2016.00160 27446806 PMC4923066

[17] Liu Y Wu L Tong R Yang F Yin L Li M . PD-1/PD-L1 inhibitors in cervical cancer. Front Pharmacol. (2019) 10:65. doi: 10.3389/fphar.2019.00065 30774597 PMC6367228

[18] Xie Y Kong W Zhao X Zhang H Luo D Chen S . Immune checkpoint inhibitors in cervical cancer: Current status and research progress. Front Oncol. (2022) 12:984896. doi: 10.3389/fonc.2022.984896 36387196 PMC9647018

[19] Tamarelle J Thiebaut ACM de Barbeyrac B Bebear C Ravel J Delarocque-Astagneau E . The vaginal microbiota and its association with human papillomavirus, Chlamydia trachomatis, Neisseria gonorrhoeae and Mycoplasma genitalium infections: a systematic review and meta-analysis. Clin Microbiol Infect. (2019) 25:35–47. doi: 10.1016/j.cmi.2018.04.019 29729331 PMC7362580

[20] Liu YR Wang SZ Liu J Su MR Diao XL Liang XL . Characteristics of vaginal microbiota in various cervical intraepithelial neoplasia: a cross-sectional study. J Trans Med. (2023) 21:816. doi: 10.1186/s12967-023-04676-5 PMC10652498 37974192

[21] Mitra A MacIntyre DA Marchesi JR Lee YS Bennett PR Kyrgiou M . The vaginal microbiota, human papillomavirus infection and cervical intraepithelial neoplasia: what do we know and where are we going next? Microbiome. (2016) 4:58. doi: 10.1186/s40168-016-0203-0 27802830 PMC5088670

[22] Lebeau A Bruyere D Roncarati P Peixoto P Hervouet E Cobraiville G . HPV infection alters vaginal microbiome through down-regulating host mucosal innate peptides used by Lactobacilli as amino acid sources. Nat Commun. (2022) 13:1076. doi: 10.1038/s41467-022-28724-8 35228537 PMC8885657

[23] Wu M Li H Yu H Yan Y Wang C Teng F . Disturbances of vaginal microbiome composition in human papillomavirus infection and cervical carcinogenesis: a qualitative systematic review. Front Oncol. (2022) 12:941741. doi: 10.3389/fonc.2022.941741 35903684 PMC9316588

[24] Herweijer E Hu K Wang J Lu D Sparén P Adami HO . Incidence of oncogenic HPV infection in women with and without mental illness: A population-based cohort study in Sweden. PloS Med. (2024) 21:e1004372. doi: 10.1371/journal.pmed.1004372 38527071 PMC11259452

[25] Li M Zeng Z Feng H Cao Y Zhang Q Lv T . Accurate 16S absolute quantification sequencing revealed vaginal microecological composition and dynamics during mixed vaginitis treatment with Fufang FuRong effervescent suppository. Front Cell Infect Microbiol. (2022) 12:883798. doi: 10.3389/fcimb.2022.883798 35646743 PMC9136393

[26] Bommana S Hu YJ Kama M Wang R Kodimerla R Jijakli K . Unique microbial diversity, community composition, and networks among Pacific Islander endocervical and vaginal microbiomes with and without Chlamydia trachomatis infection in Fiji. mBio. (2024) 15:e0306323. doi: 10.1128/mbio.03063-23 38117091 PMC10790706

[27] da Silva CS Adad SJ de Souza MAH Barcelos ACM Terra APS Murta EFC . Increased frequency of bacterial vaginosis and Chlamydia trachomatis in pregnant women with human papillomavirus infection. Gynecol Obstet Invest. (2004) 58:189–93. doi: 10.1159/000079822 15256825

[28] Dong BH Huang YX Cai HN Chen YJ Li Y Zou HC . Prevotellaas the hub of the cervicovaginal microbiota affects the occurrence of persistent human papillomavirus infection and cervical lesions in women of childbearing age via host NF-κB/C-myc. J Med Virol. (2022) 94:5519–34. doi: 10.1002/jmv.28001 35835717

[29] Huang J Yin CS Wang JL . Relationship between vaginal microecological changes and oncogene E6/E7 and high-risk human papillomavirus infection. J Obstet Gynaecol. (2023) 43:2161349. doi: 10.1080/01443615.2022.2161349 36645341

[30] Lin WY Zhang QY Chen YJ Dong BH Xue HF Lei HF . Changes of the vaginal microbiota in HPV infection and cervical intraepithelial neoplasia: a cross-sectional analysis. Sci Rep. (2022) 12:2812. doi: 10.1038/S41598-022-06731-5 35181685 PMC8857277

[31] Liu JH Liu WW Liu Y Zhou XZ Zhang ZJ Sun ZR . Prevalence of microorganisms co-infections in human papillomaviruses infected women in Northern China. Arch Gynecol Obstet. (2016) 293:595–602. doi: 10.1007/s00404-015-3826-7 26280325

[32] Liu YJ Li TY Guo RC Chen TT Wang SM Wu DK . The vaginal microbiota among the different status of human papillomavirus infection and bacterial vaginosis. J Med Virol. (2023) 95:e28595. doi: 10.1002/jmv.28595 36811337

[33] Lu H Jiang PC Zhang XD Hou WJ Wei ZH Lu JQ . Characteristics of bacterial vaginosis infection in cervical lesions with high risk human papillomavirus infection. Int J Clin Exp Med. (2015) 8:21080–8. PMC4723884 26885039

[34] Lv PP Zhao F Xu XQ Xu J Wang Q Zhao Z . Correlation between common lower genital tract microbes and high-risk human papillomavirus infection. Can J Infect Dis Med Microbiol. (2019) 2019:9678104. doi: 10.1155/2019/9678104 31885754 PMC6893239

[35] Murta EF Souza MA Araujo Junior E Adad SJ . Incidence of Gardnerella vaginalis, Candida sp and human papilloma virus in cytological smears. Sao Paulo Med J. (2000) 118:105–8. doi: 10.1590/s1516-31802000000400006 PMC11173292 10887386

[36] Verteramo R Pierangeli A Mancini E Calzolari E Bucci M Osborn J . Human Papillomaviruses and genital co-infections in gynaecological outpatients. BMC Infect Dis. (2009) 9:16. doi: 10.1186/1471-2334-9-16 19216747 PMC2656516

[37] Xu CY Zhang WY Wu MH Zhang SW . Prevalence and risk factors of lower genital tract infections among women in Beijing, China. J Obstet Gynaecol Res. (2012) 38:310–5. doi: 10.1111/j.1447-0756.2011.01624.x 21827575

[38] Zhang D Li T Chen L Zhang XS Zhao GL Liu ZH . Epidemiological investigation of the relationship between common lower genital tract infections and high-risk human papillomavirus infections among women in Beijing, China. PloS One. (2017) 12:e0178033. doi: 10.1371/journal.pone.0178033 28531212 PMC5439700

[39] Zhang Z Li T Zhang D Zong XN Bai HH Bi H . Distinction between vaginal and cervical microbiota in high-risk human papilloma virus-infected women in China. BMC Microbiol. (2021) 21:90. doi: 10.1186/s12866-021-02152-y 33765914 PMC7993496

[40] Chen X He H Xiao Y Hasim A Yuan J Ye M . CXCL10 produced by HPV-positive cervical cancer cells stimulates exosomal PDL1 expression by fibroblasts via CXCR3 and JAK-STAT pathways. Front Oncol. (2021) 11:629350. doi: 10.3389/fonc.2021.629350 34422627 PMC8377428

[41] Cheng H Dong Y Wang L Zhao X Zhe X Li D . Analysis of human papillomavirus type 16 E4, E5 and L2 gene variations among women with cervical infection in Xinjiang, China. BMC Med Genomics. (2024) 17:179. doi: 10.1186/s12920-024-01926-3 38965538 PMC11225290

[42] Fu Y Cao R Schafer M Stephan S Braspenning-Wesch I Schmitt L . Expression of different L1 isoforms of Mastomys natalensis papillomavirus as mechanism to circumvent adaptive immunity. Elife. (2020) 9:e57626. doi: 10.7554/eLife.57626 32746966 PMC7402679

[43] Song Y Wu X Xu Y Zhu J Li J Zou Z . HPV E7 inhibits cell pyroptosis by promoting TRIM21-mediated degradation and ubiquitination of the IFI16 inflammasome. Int J Biol Sci. (2020) 16:2924–37. doi: 10.7150/ijbs.50074 PMC7545706 33061806

[44] Smahel M Tejklova P Smahelova J Polakova I Mackova J . Mutation in the immunodominant epitope of the HPV16 E7 oncoprotein as a mechanism of tumor escape. Cancer Immunol Immunother. (2008) 57:823–31. doi: 10.1007/s00262-007-0418-9 PMC11030076 17962940

[45] Zhang Z Zhang D Xiao BB Zhang R Bai HH Dong HY . Primary study on the relationship between high-risk HPV infection and vaginal cervical microbiota. Zhonghua Fu Chan Ke Za Zhi. (2018) 53:471–80. doi: 10.3760/cma.j.issn.0529-567x.2018.07.006 30078257

[46] Guo YI You K Qiao J Zhao YM Geng L . Bacterial vaginosis is conducive to the persistence of HPV infection. Int J Std AIDS. (2012) 23:581–4. doi: 10.1258/ijsa.2012.011342 22930296

[47] Patrignani F Siroli L Parolin C Serrazanetti DI Vitali B Lanciotti R . Use of Lactobacillus crispatus to produce a probiotic cheese as potential gender food for preventing gynaecological infections. PloS One. (2019) 14:e0208906. doi: 10.1371/journal.pone.0208906 30625157 PMC6326422

[48] Abraham E Fairley CK Denham I Bradshaw CS Farquharson RM Vodstrcil LA . Positivity and risk factors for Trichomonas vaginalis among women attending a sexual health clinic in Melbourne 2006 to 2019. Sexually Transmitted Dis. (2022) 49:762–8. doi: 10.1097/Olq.0000000000001690 PMC9553257 35948300

[49] Seña AC Goldstein LA Ramirez G Parish AJ McClelland RS . Bacterial vaginosis and its association with incident Trichomonas vaginalis infections: a systematic review and meta-analysis. Sexually Transmitted Dis. (2021) 48:E192–201. doi: 10.1097/Olq.0000000000001537 PMC8594503 34433796

[50] Ogah CO Anikwe CC Ajah LO Ikeotuonye AC Lawani OL Okorochukwu BC . Preoperative vaginal cleansing with chlorhexidine solution in preventing post-cesarean section infections in a low resource setting: A randomized controlled trial. Acta Obstet Gynecol Scand. (2021) 100:694–703. doi: 10.1111/aogs.14060 33351989

[51] Gonçalves-Nobre JG Matos A Carreira M Santos AC Veiga LC Ginete C . The interplay between HPV, other Sexually Transmissible Infections and genital microbiome on cervical microenvironment (MicroCervixHPV study). Front Cell Infect Microbiol. (2023) 13:1251913. doi: 10.3389/fcimb.2023.1251913 38532749 PMC10963500

[52] Wang H Ma Y Li R Chen X Wan L Zhao W . Associations of cervicovaginal Lactobacilli with high-risk human papillomavirus infection, cervical intraepithelial neoplasia, and cancer: A systematic review and meta-analysis. J Infect Dis. (2019) 220:1243–54. doi: 10.1093/infdis/jiz325 31242505

[53] Armstrong E Kaul R Cohen CR . Optimizing the vaginal microbiome as a potential strategy to reduce heterosexual HIV transmission. J Intern Med. (2023) 293:433–44. doi: 10.1111/joim.13600 36544257

[54] Speetjens FM Welters MJP Slingerland M van Poelgeest MIE de Vos van Steenwijk PJ Roozen I . Intradermal vaccination of HPV-16 E6 synthetic peptides conjugated to an optimized Toll-like receptor 2 ligand shows safety and potent T cell immunogenicity in patients with HPV-16 positive (pre-)malignant lesions. J Immunother Cancer. (2022) 10:e005016. doi: 10.1136/jitc-2022-005016 36261215 PMC9582304

[55] Das P Swain T Mohanty JR Sinha S Padhi B Torondel B . Higher vaginal pH in infection with intermediate Nugent score in reproductive-age women-a hospital-based cross-sectional study in Odisha, India. Parasitol Res. (2018) 117:2735–42. doi: 10.1007/s00436-018-5962-z 29936622

[56] Mitchell C Fredricks D Agnew K Hitti J . Hydrogen peroxide-producing Lactobacilli are associated with Lower Levels of vaginal interleukin-1β, independent of bacterial vaginosis. Sexually Transmitted Dis. (2015) 42:358–63. doi: 10.1097/Olq.0000000000000298 PMC4520248 26222747

[57] Hamar B Teutsch B Hoffmann E Hegyi P Váradi A Nyirády P . Trichomonas vaginalis infection is associated with increased risk of cervical carcinogenesis: A systematic review and meta-analysis of 470 000 patients. Int J Gynecol Obstet. (2023) 163:31–43. doi: 10.1002/ijgo.14763 37010897

[58] Lazenby GB Taylor PT Badman BS Mchaki E Korte JE Soper DE . An association between Trichomonas vaginalis and high-risk human papillomavirus in Rural Tanzanian women undergoing cervical cancer screening. Clin Ther. (2014) 36:38–45. doi: 10.1016/j.clinthera.2013.11.009 24417784

[59] Zhang Z Yang Y Zhang L Wu Y Jia P Ma Q . Relationship between cervicovaginal microecological changes and HPV16/18 infection and cervical cancer in women of childbearing age. Ann Clin Lab Sci. (2023) 53:825–34. 38182150

[60] Liao Q Zhang XF Mi X Jin F Sun HM Wang QX . Influence of group B streptococcus and vaginal cleanliness on the vaginal microbiome of pregnant women. World J Clin cases. (2022) 10:12578–86. doi: 10.12998/wjcc.v10.i34.12578 PMC9791520 36579104

[61] Kusakabe M Taguchi A Sone K Mori M Osuga Y . Carcinogenesis and management of human papillomavirus-associated cervical cancer. Int J Clin Oncol. (2023) 28:965–74. doi: 10.1007/s10147-023-02337-7 PMC10390372 37294390

[62] Huang RJ Liu ZM Sun TS Zhu L . Cervicovaginal microbiome, high-risk HPV infection and cervical cancer: Mechanisms and therapeutic potential. Microbiological Res. (2024) 287:127857. doi: 10.1016/j.micres.2024.127857 39121703

[63] Giraldo PC Sanches JM Sparvolli LG Amaral R Migliorini I Gil CD . Relationship between Papillomavirus vaccine, vaginal microbiome, and local cytokine response: an exploratory research. Braz J Microbiol. (2021) 52:2363–71. doi: 10.1007/s42770-021-00616-x PMC8578365 34628621

[64] Zhang W Yin YF Jiang YS Yang YY Wang WT Wang XY . Relationship between vaginal and oral microbiome in patients of human papillomavirus (HPV) infection and cervical cancer. J Trans Med. (2024) 22:396. doi: 10.1186/s12967-024-05124-8 PMC11059664 38685022

